# Identification of the emerging fungal pathogens in Brazilian children infected by *Giardia lamblia*

**DOI:** 10.3389/fcimb.2025.1667510

**Published:** 2025-12-12

**Authors:** Carolline Soares Motta, Maria Fantinatti, Barbara de Oliveira Baptista, Gisela Lara da Costa, Wilson Savino, Alda Maria Da-Cruz, Manoel Marques Evangelista Oliveira

**Affiliations:** 1 Laboratório Interdisciplinar de Pesquisas Médicas, Instituto Oswaldo Cruz, Fundação Oswaldo Cruz, Rio de Janeiro, Brazil; 2 Instituto Nacional de Ciência e Tecnologia em Neuroimunomodulação, Instituto Oswaldo Cruz, Fundação Oswaldo Cruz, Rio de Janeiro, Brazil; 3 Disciplina de Parasitologia, DMIP, Faculdade de Ciências Médicas, Universidade do Estado do Rio de Janeiro, Rio de Janeiro, Brazil; 4 Laboratório de Taxonomia, Bioquímica e Bioprospecção de Fungos, Instituto Oswaldo Cruz, Fundação Oswaldo Cruz, Rio de Janeiro, Brazil; 5 Laboratório de Pesquisas sobre o Timo, Instituto Oswaldo Cruz, Fundação Oswaldo Cruz, Rio de Janeiro, Brazil

**Keywords:** *Giardia lamblia*, preschoolers, fungal microbiota, intestinal pathologies, *Candida parapsilosis*

## Abstract

*Giardia lamblia* is the most prevalent intestinal protozoan in Brazilian children and has been associated with alterations in the gut microbiota. While bacterial dysbiosis in giardiasis has been well studied, little is known about the associated fungal communities. This study aimed to investigate whether *Giardia* infection predisposes preschool children to the emergence of fungal pathogens and to identify which pathogenic fungi coexist in the intestines of *Giardia*-infected preschoolers. Stool samples from children aged 1–4 years living in a low-income community in Rio de Janeiro were analyzed by PCR for *Giardia* and subjected to fungal isolation and identification using MALDI-TOF MS and ITS sequencing. Among 25 samples, 13 were *Giardia*-positive, and 10 of these harbored *Candida parapsilosis*, with co-occurrence of *C. tropicalis* and *C. krusei* in some cases. *Saccharomyces cerevisiae* was also detected. This is the first report of *C. parapsilosis*, *C. krusei*, and *S. cerevisiae* in *Giardia*-infected children. The high frequency of *C. parapsilosis* raises the possibility of a synergistic interaction between protozoan and fungal infections in vulnerable pediatric populations.

## Introduction

Intestinal parasites are part of the group of enteropathogens that can lead to malnutrition, anemia, environmental enteric dysfunction, chronic immune activation, systemic inflammation, epigenetic alterations, and disruptions in the intestinal microbiota, potentially resulting in growth delay in children (Gabain et al., 2023). *Giardia lamblia* is a globally distributed intestinal protozoan pathogen, identified in several animal species, including humans, with a greater clinical impact in young children and malnourished or immunodeficient individuals (Farthing, 1997; Ortega and Adam, 1997). Infection by this protozoan is considered one of the five most common causes of intestinal infection in children under five years of age. Although most *Giardia*-infected individuals are asymptomatic, *G. lamblia* has been linked to impaired growth in preschool-aged children (Rogawski et al., 2018) and other clinical issues. The bacterial microbiota plays a key role in immune regulation and homeostasis, and its balance is crucial for controlling *Giardia* infection (Shady et al., 2024). Although historically less explored than bacteria, the fungal community forms an essential part of the intestinal microbiota, establishing symbiotic interactions with the mammalian host under conditions of homeostasis.

On the other hand, dysbiosis can promote fungal overgrowth, with some species becoming pathogenic (Sokol et al., 2017). Growing evidence indicates that intestinal fungi play a relevant role in regulating host homeostasis, influencing immunological, physiological, and pathophysiological responses, in addition to contributing to the structuring and stability of the commensal bacterial microbiota (Underhill and Iliev, 2014; Wheeler et al., 2016). Among these, fungi of the genus *Candida* stand out, especially *C. albicans*, which has a significant impact on both the composition and functional dynamics of the bacterial microbiome through biofilm formation and metabolite exchange (Kombrink et al., 2019; Fernández de Ullivarri et al., 2020). *C. albicans* was first observed in *Giardia*-infected patients in the 1970s, but not in the control group, suggesting a possible influence of *C. albicans* on *G. lamblia* proliferation. Cheissin, 1963 (Cheissin, 1961), also reported that drugs inhibiting *C. albicans* were effective against *Giardia*, leading the authors to infer a relationship with *Giardia* pathogenesis.

*Giardia* is known to cause environmental enteric dysfunction, characterized by alterations in the intestinal barrier — including villus atrophy, inflammation, mucin disruption, pH changes, and reduced nutrient absorption — which are believed to favor the proliferation of opportunistic fungi such as *C. albicans*, *C. parapsilosis*, *C. krusei*, and *C. tropicalis* (Allain et al., 2017). Conversely, *Candida* spp. can modulate the intestinal immune response — for example, by altering cytokine production and recruiting immune cells — thereby intensifying intestinal inflammation and potentially influencing the host response against *Giardia*. However, no direct study to date has demonstrated specific competition or synergistic interactions between *Giardia* and *C. albicans*, *C. parapsilosis*, *C. krusei*, or *C. tropicalis*. Nonetheless, the academic literature remains extremely scarce regarding the relationship between *Giardia* and emerging pathogenic fungi. Herein, we evaluated whether *Giardia* infection coexists with specific fungal species in the gut.

## Materials and methods

This study involved children attending daycare in a low-income community in Rio de Janeiro, Brazil. The area is environmentally heterogeneous: the initial area is more urbanized, while the peripheral area lacks basic sanitation, including water supply, paved streets, sewage collection, and electricity. Children under five years of age attending this daycare center were included in the study. Preschool children whose stool samples were not submitted or were submitted under non-compliant conditions were excluded. Non-compliant conditions included insufficient sample volume, inadequate preservation, collection time exceeding 24 hours, or contamination with liquids (urine, toilet water, or others). Children who had recently used antibiotics (within the last 6 months) were also excluded.

Stool samples were obtained for convenience and submitted to molecular *Giardia* diagnosis by PCR, and fungal isolation and identification polyphasic taxonomy. For molecular diagnosis, stool samples had their DNA extracted using the QIAamp Fast DNA Stool Mini Kit (Qiagen GmbH, Germany) according to the manufacturer’s instructions, except for the lysis temperature, which was increased to 95°C, and the final elution volume was reduced to 100µL. PCR amplification was performed followed by nested PCR, using the *βgia* gene target (Cacciò et al., 2002; Lalle et al., 2005). Positivity was verified through 1.5% agarose gel electrophoresis.

**Figure 1:**
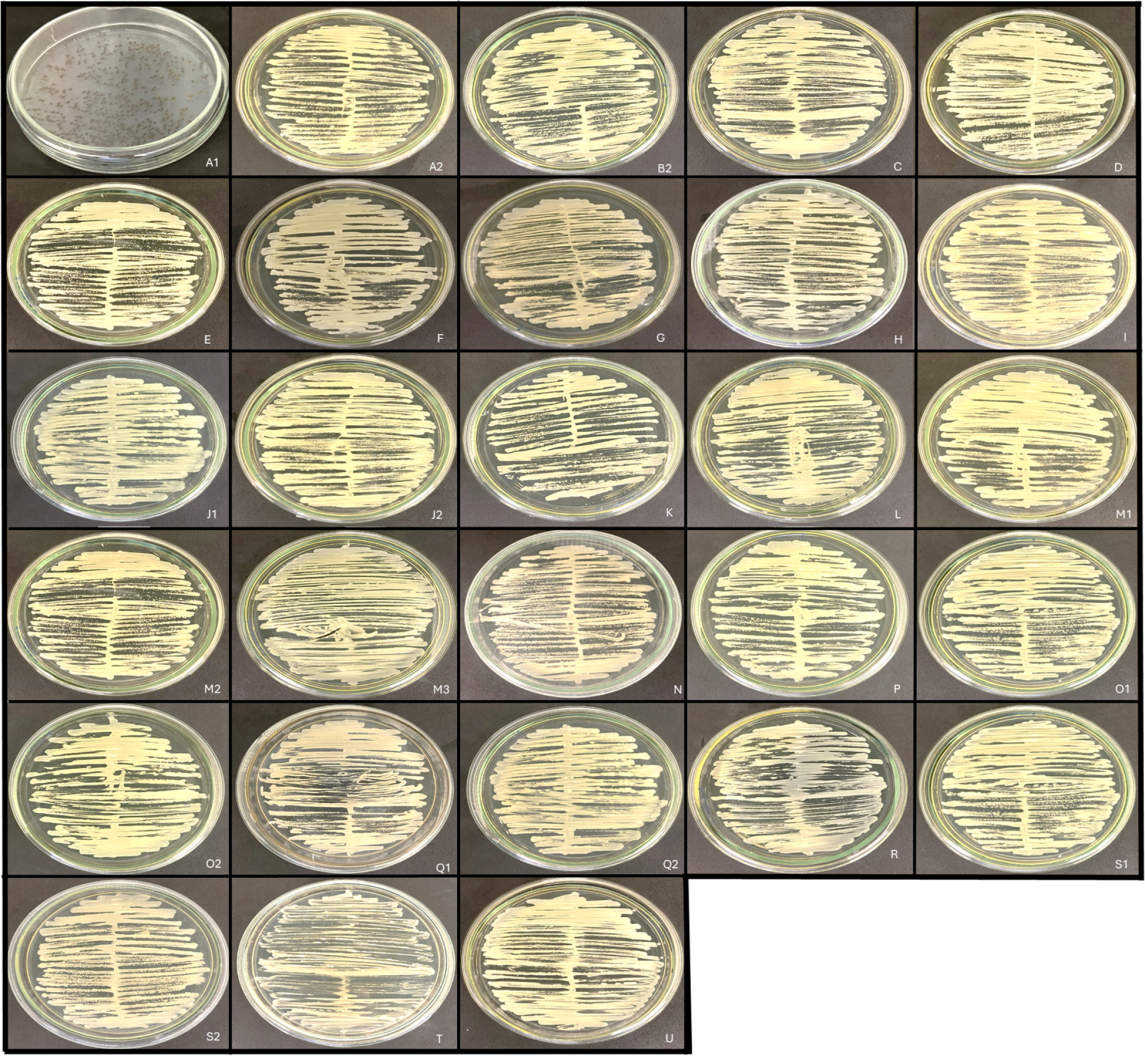
Clinical samples from children seeded in Sabouraud Dextrose Agar (SDA). Different yeast colonies were identified and isolated according to their morphological characteristics. *Candida* species were identified in the samples **A2, B1-2, C, D, E, F, G, H, I, J1-2, K, L, M1-3, N, P, O1-2, Q1-2, R, S1, 2** and **U**. Non*-Candida* species were identified in A1 and T samples (*Exophiala* sp. and *Saccharomyces cerevisiae*, respectively, were identified).

For fungal identification the samples were streaked onto Sabouraud Dextrose Agar (SDA, DIFCO, Becton-Dickinson and Company, Holdrege, Nebraska, USA) and incubated at 30°C for 48 hours to allow for morphological assessments (**Figure 1**). Colonies presenting distinct macromorphological characteristics on SDA were subcultured onto CHROMagar Candida Plus (CHROMagar™, Saint-Denis, France) for screening of *C. auris*. Colony characteristics on these selective media were then interpreted following the manufacturer’s guidelines to confirm the presence of different yeast species.

Fungal identification via MALDI-TOF MS was carried out as previously described by Pinto et al. (2022) (Pinto et al., 2022) using about 10^6^ yeast cells (~1μg) that were transferred from the culture plate (c.a. 1g) (Bruker, UK) to a 500 μl tube containing 20 μl of 70% formic acid in water (v/v) and 10 μl of acetonitrile for protein extraction. A 1 μL aliquot of this protein-containing solution was spotted onto a stainless-steel MALDI-TOF MS plate, covered with 1μL of an α-cyano-4-hydroxycinnamic acid matrix solution (CHCA, Fluka, Switzerland) and air-dried at room temperature prior to spectra acquisition. Each sample was analyzed in triplicate. Identification scores were expressed as log values ranging from 0 to 3, with values ≥1.7 considered reliable for genus-level identification and ≥2.0 for species-level identification. The ITS region sequencing was performed at the FIOCRUZ Sequencing Platform (Rio de Janeiro, Brazil). Colony PCR was performed as outlined by Corrêa-Moreira et al., 2024 (Corrêa-Moreira et al., 2024). Yeast colonies grown on SDA plates at 30°C for 48 h were used as the source of DNA. A small portion of each isolated colony was transferred with a micropipette tip directly into PCR tubes as the DNA template. Cells were lysed by heating in a microwave for 90 seconds, followed by immediate cooling on ice to prevent DNA degradation. PCR amplification was carried out in a 50 μL reaction mixture containing 25 ng of genomic DNA, 10 pmol of universal fungal primers ITS1 and ITS4, using an annealing temperature of 58°C in a 96-well thermocycler (Applied Biosystems, Thermo Fisher Scientific). The amplified products were purified with a QIAquick^®^ PCR Purification Kit (QIAGEN^®^) according to the manufacturer’s protocol. Sequences were edited using CodonCodeAligner v. 9.0.2 software and compared to NCBI GenBank entries via Basic Local Alignment Search Tool (BLAST). Phylogenetic analysis was performed using the neighbor-joining algorithm (default) of Saitou and Nei (1987) with 1000 bootstrap replicates (Felsenstein, 1985), based on the alignment of the obtained ITS sequences of reference strains belonging to different clades (South Asian, East Asian, South African, South American, and Iran) deposited in GenBank. The tree was drawn to scale, with branch lengths in the same units as those of the evolutionary distances used to infer the phylogenetic tree. The evolutionary distances were computed using the Maximum Composite Likelihood method (Tamura et al., 2004) and are in the units of the number of base substitutions per site. Codon positions included were 1st+2nd+3rd+Noncoding. All positions containing gaps and missing data were eliminated from the dataset (complete deletion option). Phylogenetic analyses were conducted in MEGA4 (Tamura et al., 2007).

## Results and discussion

In Brazil, *G. lamblia* is the most prevalent pathogenic intestinal protozoan, particularly among children under five years of age (Coelho et al., 2017). In this age group, behaviors such as oral exploration, developing hygiene habits, gaining mobility, and the presence of an immature immune system increase susceptibility to *G. lamblia* and other orally transmitted pathogens. This susceptibility is especially pronounced in socially and sanitary vulnerable settings, such as low-income communities, where malnutrition can further compromise the immune response to infection.

**Figure 2:**
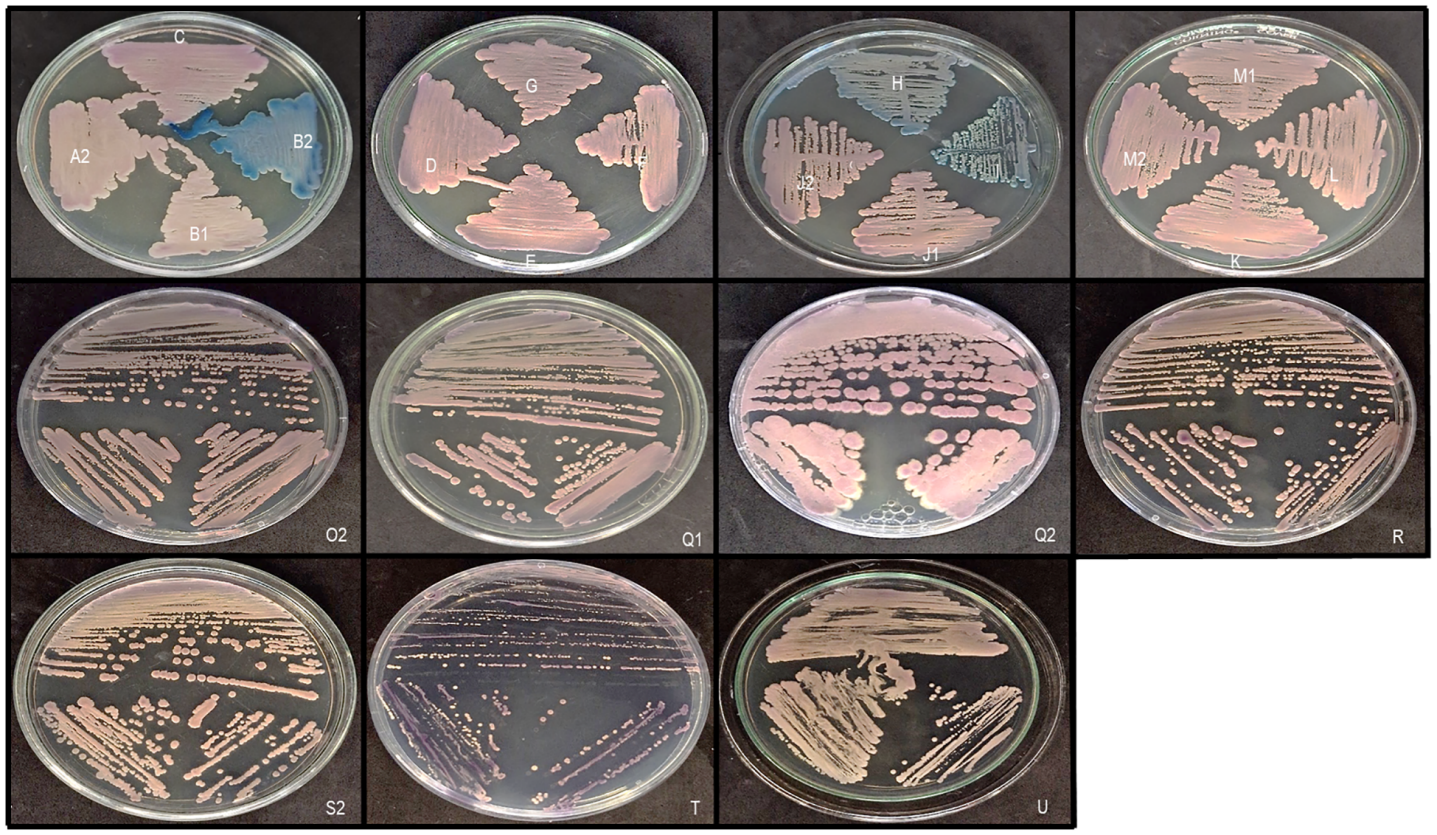
Subculture of samples in CHROMagar Candida Plus (CHROMagar™) media. The samples were subcultured in a selective and differential media, formulated to visually identify different species of candida based on colony color. Colonies of *Candida parapsilosis* were identified in samples **A2, B1, C, D, E, F, G, J1, J2, K, L, M1, M2, M3, N, O2, P, Q1, R, S2** and **U**, *C. tropicalis* in samples **B2, O1** and **S1**, *C. albicans* in samples H and I and *C. krusei* in sample Q2. *Saccharomyces cerevisiae* was also identified in sample T.

**Figure 3:**
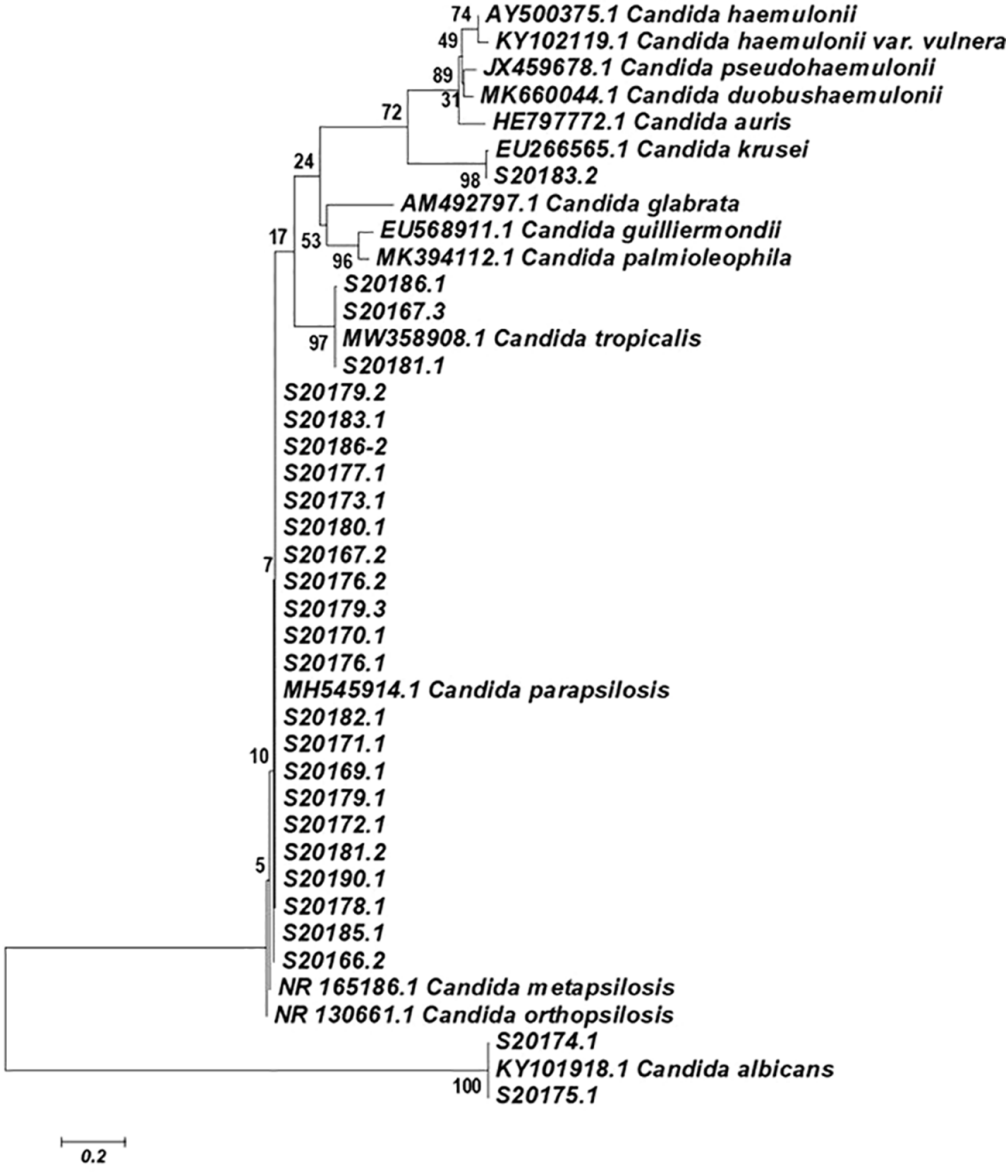
Evolutionary relationships of 41 taxa. The evolutionary history was inferred using the Neighbor-Joining method. The optimal tree with the sum of branch length = 0.05879796 is shown. The percentage of replicate trees in which the associated taxa clustered together in the bootstrap test (1000 replicates) are shown next to the branches. The tree is drawn to scale, with branch lengths in the same units as those of the evolutionary distances used to infer the phylogenetic tree. The evolutionary distances were computed using the Maximum Composite Likelihood method and are in the units of the number of base substitutions per site. Codon positions included were 1st+2nd+3rd+Noncoding. All positions containing gaps and missing data were eliminated from the dataset (Complete deletion option). There were a total of 646 positions in the final dataset. Phylogenetic analyses were conducted in MEGA4 (Tamura et al., 2007).

A total of 25 samples were analyzed, and 13 tested positive for *Giardia* genetic material. The evaluation of the fungal microbiota in these 13 preschoolers revealed that ten of them harbored *C. parapsilosis*; among them, one also presented co-occurrence with *C. tropicalis*, and another with *C. krusei* (**Table 1**; **Figure 2**; **Figure 3**).

**Table 1:** Occurrence of fungi in fecal samples from preschool children infected or not infected with *Giardia lamblia*, according to gender, age, and stool consistency.

Sample ID	Gender	Age (mos.)	Stool consistency	PCR *Giardia*	Fungal microbiota
166	M	12,68	Well-formed	–	*Exophiala sp.* and *Candida parapsilosis*
167	M	35,28	Mushy	–	*C. parapsilosis*
168	M	65,87	Dry	–	No growth
169	F	34,20	Well-formed	+	*C. parapsilosis*
170	M	27,30	Dry	–	*C. parapsilosis*
171	M	33,61	Well-formed	+	*C. parapsilosis*
172	F	43,43	Mushy	+	*C. parapsilosis*
173	M	45,3	Mushy	–	*C. parapsilosis*
174	F	18,96	Mushy	–	*C. albicans*
175	M	28,38	Mushy	–	*C. albicans*
176	F	19,68	Dry	–	*C. parapsilosis*
177	F	37,42	Mushy	–	*C. parapsilosis*
178	F	32,1	Well-formed	+	*C. parapsilosis*
179	M	37,29	Mushy	+	*C. parapsilosis*
180	F	32,10	Liquid	+	*C. parapsilosis*
181	F	18,92	Well-formed	+	*C. tropicalis and C. parapsilosis*
182	F	26,91	Mushy	–	*C. parapsilosis*
183	F	42,97	Well-formed	+	*C. parapsilosis* and *C. krusei*
184	F	25,26	Well-formed	–	No growth
185	M	17,58	Mushy	+	*C. parapsilosis*
186	M	18,86	Well-formed	–	*C. tropicalis and C. parapsilosis*
187	M	42,77	Mushy	+	No growth
188	M	37,52	Mushy	+	*Saccharomyces cerevisiae*
189	F	25,69	Well-formed	+	No growth
190	M	36,40	Mushy	+	*C. parapsilosis*

M, Male; F, Female; (-) *Giardia* negative; (+) *Giardia* positive, mos, months.

**Figure 4:**
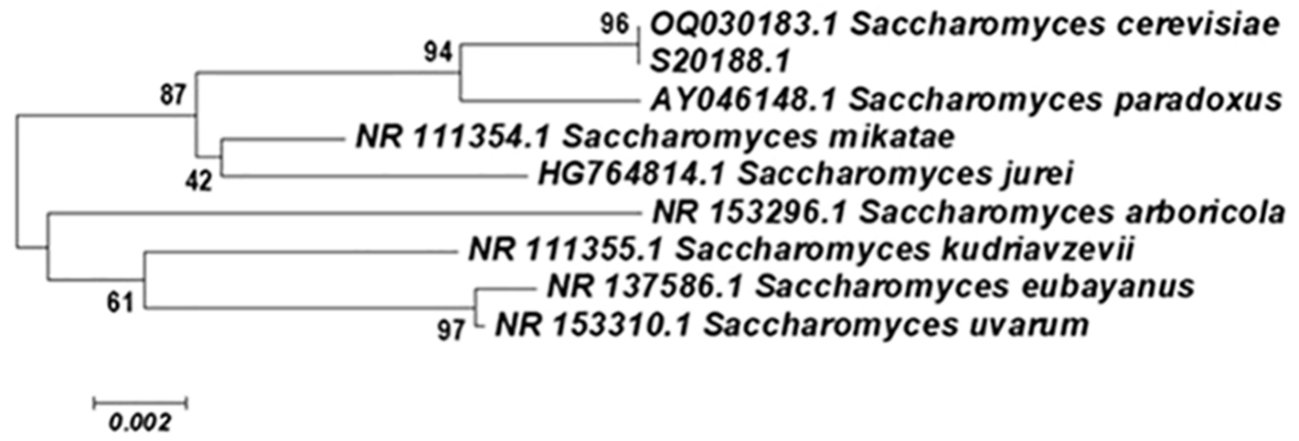
Evolutionary relationships of 9 taxa. The evolutionary history was inferred using the Neighbor-Joining method. The optimal tree with the sum of branch length = 0.05879796 is shown. The percentage of replicate trees in which the associated taxa clustered together in the bootstrap test (1000 replicates) are shown next to the branches. The tree is drawn to scale, with branch lengths in the same units as those of the evolutionary distances used to infer the phylogenetic tree. The evolutionary distances were computed using the Maximum Composite Likelihood method and are in the units of the number of base substitutions per site. Codon positions included were 1st+2nd+3rd+Noncoding. All positions containing gaps and missing data were eliminated from the dataset (Complete deletion option). There were a total of 646 positions in the final dataset. Phylogenetic analyses were conducted in MEGA4 (Tamura et al., 2007).

Regarding the remaining three *Giardia*-positive samples, one harbored *S. cerevisiae* (**Figure 2**, **Figure 4**) and two showed no growth. Despite this, our screening for *C. auris*, the main emerging pathogenic yeast (Casadevall et al., 2021), was negative. Among the samples negative for *Giardia*, *C. albicans* and *C. parapsilosis* were also present. The fungal load was estimated based on colony-forming unit counts; however, no significant differences were observed between *Giardia*-positive and -negative children (**Supplementary Figure S1**). Similarly, fungal load did not vary according to stool consistency (**Table 1**). Furthermore, *Exophiala* sp. was also identified in children, but in a *Giardia*-negative sample.

The presence of *C. albicans* and *C. tropicalis* has already been reported in *Giardia*-infected patients (Naik et al., 1978). Importantly, this is the first report of *C. parapsilosis*, *C. krusei*, and *S. cerevisiae* in individuals infected with *G. lamblia.* The high frequency of *C. parapsilosis* observed among children in this region warrants particular attention. While *C. parapsilosis* can be found in the intestinal microbiota of healthy children, its growth may be favored during dysbiosis. *C. parapsilosis* is an emerging human pathogen and is considered one of the main causes of invasive candidiasis (Daneshnia et al., 2023). Excessive intestinal growth of *C. parapsilosis* can lead to fungal translocation into the bloodstream, especially in immunocompromised or immunosuppressed patients, resulting in systemic infections such as candidemia (Trofa et al., 2008). Accordingly, *G. lamblia* infection may cause barrier dysfunction through direct damage to enterocytes and increased intestinal permeability (Cascais-Figueiredo et al., 2019), which might favor the invasive process of *C. parapsilosis* and other fungi, such as *C. albicans*, *C. tropicalis*, *C. krusei*, and *S. cerevisiae*, as observed in the present study.

The present study design did not allow us to determine whether *Giardia* infection predisposes individuals to fungal infection or vice versa. Moreover, in uncontrolled real-world settings such as the one investigated here, it is very difficult to determine which pathogen represents the primary infection and how one infection might influence the other. Stool consistency was assessed, and no additional symptoms were reported by these children. Thus, it cannot be concluded that this represented a fungal infection, but rather fungal colonization. Although the coexistence of both the protozoan and fungi in the intestine is expected to reflect intestinal dysbiosis, further studies are needed to elucidate this ecological relationship.

Another limitation of our study is the small sample size, which precluded the inclusion of controls and limited our ability to assess potential associations between fungal load and *Giardia* infection. Research conducted in hard-to-reach areas often faces methodological constraints. Because samples intended for microbiota analysis could not contain preservatives and required refrigeration, the number of participants who met the inclusion criteria was limited.

Nevertheless, despite the small sample size, it was possible to demonstrate that, regardless of the presence of *G. lamblia*, fungi such as *S. cerevisiae*, *C. albicans*, *C. parapsilosis*, *C. krusei*, and *C. tropicalis* could still be identified.

The coexistence of *G. lamblia* and *S. cerevisiae* had not been previously reported, although *in vitro*, in addition to classical endocytosis, *Giardia* is capable of internalizing this yeast, suggesting that it might serve as a nutritional source for the protozoan (Benchimol, 2021). Furthermore, in recent years, *S. cerevisiae* has been reported as an emerging pathogen associated with death in Brazil (Ramos et al., 2023).

In this study, it was not possible to establish a statistically significant association between the presence of *Giardia* and pathogenic fungi. However, it is well established that the relationship between parasitic infections and the composition of the intestinal microbiota is bidirectional: an unbalanced microbiota can favor parasite colonization, whereas its restoration can contribute to infection control (Pryshliak et al., 2022; Shady et al., 2024).

## Final remarks

Herein, in addition to *C. albicans* and *C. tropicalis*, we identified the presence of *C. parapsilosis*, *C. krusei*, and *S. cerevisiae* in children infected with *G. lamblia*. Although the small sample size does not allow us to establish an association between the presence of the protozoan and the respective fungi, it supports the high occurrence of this coexistence, which had not been previously reported. This finding opens an avenue for further studies investigating this coexistence in terms of: (1) the biological characteristics of the pathogens, such as proliferation; (2) ecology and population dynamics; and (3) pathogenesis, immune response modulation, and symptom manifestation in parasitized individuals.

## Data Availability

The datasets presented in this study can be found in online repositories. The names of the repository/repositories and accession number(s) can be found in the article/**Supplementary Material**.

## Supplementary material

Supplementary Figure 1 Estimated fungal load in preschoolers parasitized by *Giardia*. Fungal load was assessed based on colony-forming unit (CFU) counts. Each symbol represents an individual participant: blue circles indicate Giardia-negative preschoolers, and red triangles indicate Giardia-positive preschoolers. Horizontal bars represent median values. P value: 0,95.
