# Supplementary figures and images for: Identification of the emerging fungal pathogens in Brazilian children infected by Giardia lamblia
Source: Front Cell Infect Microbiol. 2025 Dec 12;15:1667510. doi: 10.3389/fcimb.2025.1667510 (PMC12741149; doi:10.3389/fcimb.2025.1667510)

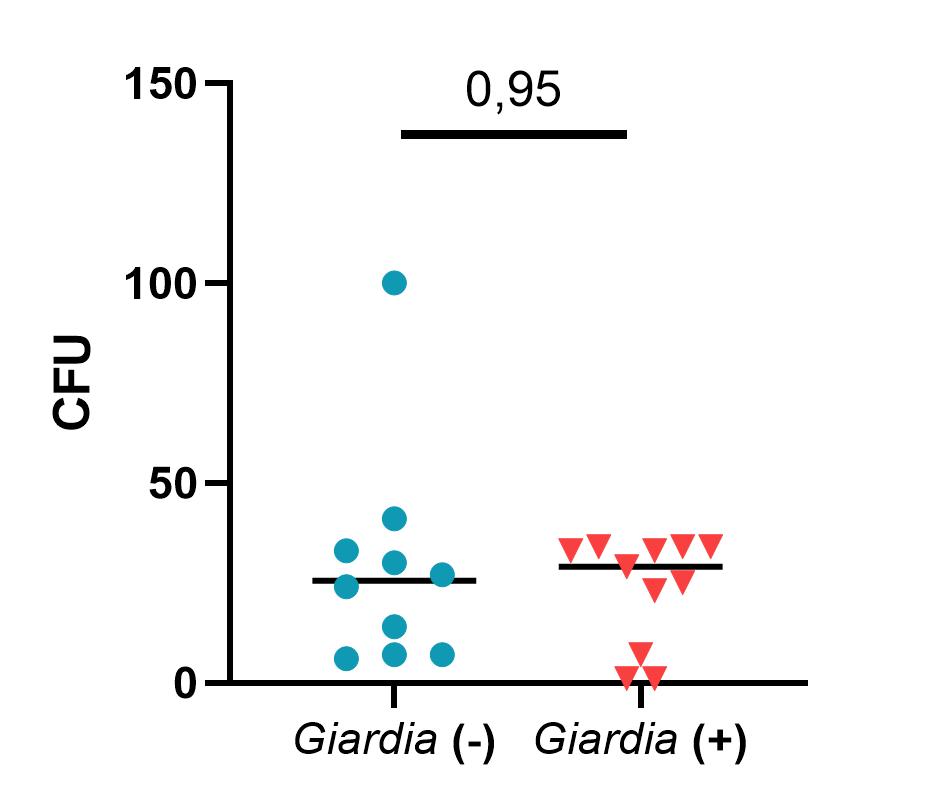

Supplement: Supplementary Figure 1 — Estimated fungal load in preschoolers parasitized by Giardia. Fungal load was assessed based on colony-forming unit (CFU) counts. Each symbol represents an individual participant: blue circles indicate Giardia-negative preschoolers, and red triangles indicate Giardia-positive preschoolers. Horizontal bars represent median values. P value: 0,95. [file Image1.jpeg]
